# Robotic-assisted Laparoscopic Ureterocalicostomy (RALUC): How we do it

**DOI:** 10.1590/S1677-5538.IBJU.2025.0125

**Published:** 2025-03-29

**Authors:** Jens-Uwe Stolzenburg, Doreen Trebst, Theodoros Spinos, Toni Franz, Anja Dietel, Stefan Siemer, Matheus Miranda Paiva, Evangelos Liatsikos, Ho Thi Phuc

**Affiliations:** 1 University of Leipzig Department of Urology Leipzig Germany Department of Urology, University of Leipzig, Leipzig, Germany; 2 University of Patras Department of Urology Patras Greece Department of Urology, University of Patras, Patras, Greece; 3 University of Saarland Department of Urology Homburg Germany Department of Urology, University of Saarland, Homburg, Germany; 4 Hospital Irmandade da Santa Casa da Misericórdia de Santos Serviço de Urologia Santos SP Brasil Serviço de Urologia, Hospital Irmandade da Santa Casa da Misericórdia de Santos, Santos, SP, Brasil

## Abstract

**Purpose:**

Ureterocalicostomy refers to the anastomosis of the lower pole calyces with the ureter after excision of the hydronephrotic lower renal pole (1, 2). Indications for ureterocalicostomy include previous failed pyeloplasty, ureteropelvic junction obstruction (UPJO) with anatomical abnormalities, such as intrarenal pelvis or short ureter (3) and proximal ureteral strictures (4). The purpose of this video is to demonstrate the technique of Robotic-Assisted Laparoscopic Ureterocalicostomy (RALUC) in a patient with UPJO and intrarenal pelvis.

**Materials and Methods:**

Preoperatively, a retrograde ureteropyelography was performed. A transperitoneal approach with the Hassan technique was used, followed by the introduction of four additional DaVinci® trocars. The first step of the procedure is dissection of the retroperitoneum, the proximal ureter and lower part of the kidney including the renal hilum. The proximal ureter is dissected below the stricture. The lower pole artery is selectively bulldogged, and the lower pole of the kidney is resected in a circular manner to get broad based access to the lowest calix. The "Garland" suture technique is used to control hemostasis of the lower pole of the kidney. Therefore, a running, "low tension", circular suture is performed along the whole renal defect. This provides sufficient parenchymal hemostasis without narrowing the access to the lower calix. The ureter is then spatulated and sutured to the lower calix. The video shows step by step the ureterocalical anastomosis in single knot technique and explains tips and tricks.

**Results:**

Total operative time was 114 minutes, while estimated blood loss was 25 mL. The JJ catheter was removed at 40 days postoperatively, while an ultrasound was performed after the JJ removal, showing no hydronephrosis. No intraoperative or postoperative complications were reported. The creatinine count and GFR after JJ removal were 92 μmoL/L and 70 ml/min, respectively. During the last follow-up the patient remained asymptomatic and had a mild chronical dilatation of the caliceal system but no hydronephrosis.

**Conclusions:**

This video demonstrates the effectiveness and repeatability of RALUC for reconstructing UPJO in patients with very narrow or intrarenal pelvis. RALUC is a feasible, safe and efficient approach for selected patients requiring reconstruction of the upper urinary tract.

Available at: http://www.intbrazjurol.com.br/video-section/20250125_Stolzenburg_et_al

**Video 1:** 
